# Treatment of *Pneumocystis* pneumonia with intermediate-dose and step-down to low-dose trimethoprim–sulfamethoxazole: lessons from an observational cohort study

**DOI:** 10.1007/s15010-015-0851-1

**Published:** 2015-10-15

**Authors:** Dina Creemers-Schild, Frank P. Kroon, Ed. J. Kuijper, Mark G. J. de Boer

**Affiliations:** Department of Infectious Diseases, Leiden University Medical Center, PO Box 9600, 2300 RC Leiden, The Netherlands; Department of Medical Microbiology, Leiden University Medical Center, Leiden, The Netherlands

**Keywords:** *Pneumocystis jirovecii*, Treatment, Low dose, Cotrimoxazole, Trimethoprim–sulfamethoxazole, Antibiotic stewardship

## Abstract

**Background:**

The recommended treatment of *Pneumocystis jirovecii* pneumonia (PCP) is high-dose trimethoprim–sulfamethoxazole (TMP–SMX) in an equivalent of TMP 15–20 mg/kg/day and SMX 75–100 mg/kg/day for 2 or 3 weeks. High rates of adverse events are reported with this dose, which raises the question if lower doses are possible.

**Methods:**

All adult patients diagnosed with PCP in various immune dysfunctions and treated with TMP–SMX between January 1, 2003 and July 1, 2013 in a tertiary university hospital were included. Per institutional protocol, patients initiated treatment on intermediate-dose TMP–SMX (TMP 10–15 mg/kg/day) and could be stepped down to low-dose TMP–SMX (TMP 4–6 mg/kg/day) during treatment. Clinical variables at presentation, relapse rate and mortality rates were compared between intermediate- and step-down treatment groups by uni- and multivariate analyses.

**Results:**

A total of 104 patients were included. Twenty-four patients (23 %) were switched to low-dose TMP–SMX after a median of 4.5 days (IQR 2.8–7.0 days). One relapse (4 %) occurred in the step-down group versus none in the intermediate-dose group. The overall 30-day mortality was 13 %. There was 1 death in the step-down group (4 %) compared to 13 deaths (16 %) in the intermediate-dose group.

**Conclusions:**

We observed high cure rates of PCP by treatment with intermediate-dose TMP–SMX. In addition, a step-down strategy to low-dose TMP–SMX during treatment in selected patients appears to be safe and does not compromise the outcome of treatment.

## Introduction

According to current guidelines and reviews, the recommended treatment of *Pneumocystis jirovecii* pneumonia (PCP) is high-dose trimethoprim–sulfamethoxazole (TMP–SMX) in a dose of TMP 15–20 mg/kg/day and SMX 75–100 mg/kg/day for a duration of 2 or 3 weeks [1–3]. Most, if not all, of the evidence for this regimen is based on animal studies [4] and a small open label trial comparing high- versus low-dose TMP–SMX for the treatment of PCP in immunocompromised children [5]. In this clinical study performed almost 40 years ago, numerically more patients recovered on the high-dose regimen of TMP 20 mg/kg/day–SMX 100 mg/kg/day (11 out of 14) than when receiving TMP 4–7 mg/kg/day–SMX 20–35 mg/kg/day (3 out of 6). In randomized trials comparing different therapeutic options for the treatment of HIV-associated PCP, high-dose TMP–SMX was always present as one of both treatment arms [6–10]. Irrespective of the response rates on high-dose TMP–SMX, high rates of adverse events and drug toxicity were observed. Rash, drug fever, neutropenia, renal insufficiency, electrolyte disorders and liver toxicity were all encountered frequently. In the above-named trials, the severity of the side effects forced clinicians to switch to an alternative treatment (like pentamidine, dapsone–trimethoprim or clindamycin–primaquine) in over 30 % of patients that were included in the high-dose TMP–SMX arms.

Limited retrospective data and one small randomized trial demonstrated that treatment of PCP with an intermediate dose of TMP–SMX (TMP 10 mg/kg/day and SMX 50 mg/kg/day) yielded comparable efficacy as the traditional high-dose regimen [11, 12]. Moreover, the intermediate dose was associated with lower rate of observed toxicity and treatment limiting adverse effects. The efficacy of treatment with low-dose TMP–SMX (TMP 4–6 mg/kg/day and SMX 20–30 mg/kg/day) has not been investigated in a clinical setting ever since the first introduction of TMP–SMX for the treatment of PCP by Hughes et al. in 1975. However, the safety and comparable efficacy of a step-down strategy to low-dose TMP–SMX after observation of adverse events was demonstrated in a small prospective study in 1990 [13].

Regarding the limited evidence from clinical studies as well as the high burden of drug toxicity and treatment limiting adverse events, rationale is provided for movement away from the generally recommended high-dose TMP–SMX regimen. Since several years we routinely used in our institution an intermediate dose of TMP–SMX for all patients diagnosed with PCP. After starting this intermediate-dose regimen, it is an option for the attending clinician to switch to low-dose TMP–SMX based on observation of good clinical response or drug-related toxicity. In this study, we present the outcomes of this alternative treatment strategy. In addition, we assessed whether clinical characteristics at presentation may be indicative for later switching to low-dose TMP–SMX. In Table 1, an overview of the different dosing regimens referred to in this article is provided.

**Table 1:** Description of different TMP–SMX doses

	TMP (mg/kg/day)	SMX (mg/kg/day)	Standard dose used in the study
High dose	15–20	75–100	Not used
Intermediate dose	10–15	50–75	2400 mg b.i.d.
Low dose	4–6	20–30	960 mg b.i.d.

## Methods

### Setting and study population

The study was performed in the Leiden University Medical Center, a tertiary care and teaching hospital in the Netherlands with solid organ and haematological transplantation programs as well as providing care for HIV-seropositive patients. The study was approved by the ethics committee of the Leiden University Medical Center. Written informed consent was not obtained, since patient information was anonymized and the methods of the study did not require informed consent under common law and ethics.

The databases of the Department of Medical Microbiology and the Pharmacy Department were cross-linked to identify all adult patients diagnosed with PCP and treated with TMP–SMX between January 1, 2003 and July 1, 2013. A confirmed diagnosis of PCP was defined as presence of respiratory symptoms, radiological evidence (interstitial pneumonitis on chest X-ray or computed tomography) and positive microbiological test, i.e. a positive polymerase chain reaction (PCR) and/or positive microscopy for *P. jirovecii* cysts by silver and Giemsa staining in bronchoalveolar lavage (BAL) fluid or induced sputum. Except for the implementation of a real-time-PCR method for detection of the dihydropteroate synthase (DHPS) coding region of *P. jirovecii* DNA in 2004 [14], the diagnostic and treatment protocols for PCP did not change during the study period. Confirmed cases were included in a retrospective cohort study. Patients that were <18 years old or who were treated with other regimens active against *P. jirovecii* were excluded from the analyses. Relapse of PCP was defined as a new diagnosis of PCP within 30 days after completion of at least a 2-week course of treatment after which clinical cure was achieved. In case of relapse, only the first episode of PCP was included.

### Treatment conditions

Initial, protocolled treatment for all patients diagnosed with PCP consisted of intermediate-dose TMP–SMX administered by oral or intravenous route twice daily. In Table 1, the different dosing regimens are specified. The standard duration of treatment was 2 weeks. Additional steroid therapy was started if the partial oxygen pressure was <9.0 kPa. After commencing treatment with the intermediate dose, a step-down strategy to low-dose TMP–SMX could be followed as per decision of the attending physician, usually after consultation with an infectious disease specialist or microbiologist. The decision would be conditional, based on observation of a favourable clinical response to initial therapy or in case of development of toxicity.

The intermediate-dose group consisted of patients who received at least 2 weeks of the intermediate dose of TMP–SMX. Patients who were switched to low-dose TMP–SMX during treatment were included in the step-down group.

### Data collection and analyses

For all included patients, clinical data about underlying disease, symptoms and laboratory values at presentation were extracted from the electronic patient files. Because the assay used to measure lactate dehydrogenase (LDH) levels was altered during the study period, we defined an elevated LDH as more than 1.5 times the upper limit of normal. Data concerning the diagnostic workup for PCP, the occurrence of ICU admission, length of hospitalization, 30-day relapse rate of PCP, 30- and 100-day all-cause mortality and recorded adverse events were documented.

Variables were compared between treatment groups. In addition, the 30-day mortality data were analysed in a case-by-case review. The cause of death was classified as either attributable to PCP (e.g. respiratory failure), contributable to PCP (e.g. secondary bacterial sepsis) or not related to PCP (e.g. gastrointestinal bleeding, myocardial infarction).

Statistical analyses were performed with the SPSS version 20.0 statistical package for Windows. Univariate risk factor analysis for binominal variables was performed using cross tables and Chi square statistical tests. Results are reported as risk ratios (RR) with 95 % confidence intervals (CI95 %). For continuous variables, a Student’s *t* test or Mann–Whitney *U* test was performed depending on the distribution of the respective variable. A *p* value of <0.05 was considered to be significant. The observational study design leads to an inevitable confounding by indication. The effects of this bias and interpretation of results are discussed in detail in “Discussion”.

## Results

### Study cohort

**Fig. 1:**
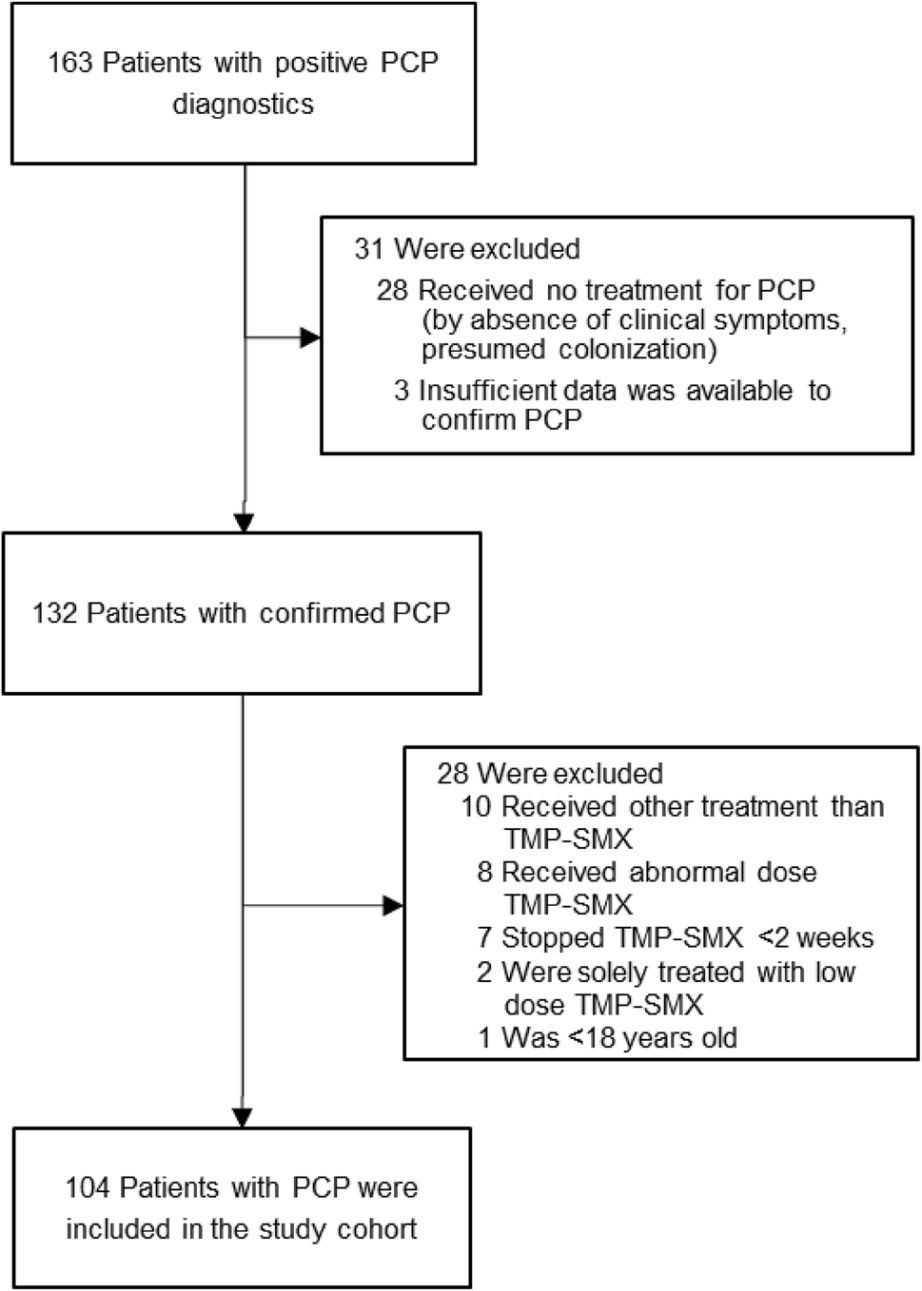
The study cohort

Out of 163 potential cases of PCP retrieved by query from the databases of the departments of medical microbiology and pharmacy, 132 patients with a confirmed diagnosis of PCP were identified. Ten patients were treated with a regimen other than TMP–SMX, eight patients received a dose of TMP–SMX that could not be classified per protocol in one of the two defined treatment groups and two patients were solely treated with low-dose TMP–SMX. One patient was a child. Seven patients prematurely terminated treatment before the end of 2 weeks. For five patients, this was due to toxicity and for two patients the reason was unknown. A total of 104 adult patients were included in the study cohort (Fig. 1).

Eighty patients (77 %) received the standard treatment of at least 2 weeks of intermediate-dose TMP–SMX. The majority of these patients (73 patients, 91 %) were treated for 2 weeks. Six patients were treated for 3 weeks and one patient was treated for 4 weeks. Twenty-four patients of the study cohort (23 %) were included in the step-down group. They were switched to low-dose TMP–SMX at a median of 4.5 days (IQR 2.8–7.0 days). In the whole study cohort, only one patient used TMP–SMX prophylaxis (480 mg o.d.) prior to diagnosis of PCP. This patient was recently diagnosed with HIV infection and had started taking prophylaxis only 15 days prior to the diagnosis of PCP.

Respiratory material was obtained from all patients as required by definition for a confirmed diagnosis of PCP. A BAL was performed in 99 (95 %) of the cases. In the remaining five patients, two patients in the step-down group and three patients in the intermediate-dose group, diagnostic tests were performed on sputum. Microscopy for *P. jirovecii* cysts was positive in 14 patients (70 %) in the step-down group and in 41 patients (57 %) in the intermediate-dose group.

### Clinical characteristics at presentation

The clinical characteristics at presentation with PCP are described in Table 2. The LDH serum level was more frequently elevated in the intermediate-dose group (*p* = 0.048). None of the other studied variables discriminated between the two treatment groups.

**Table 2:** Clinical characteristics at presentation per treatment schedule

	Step-down group (*n* = 24)	Intermediate-dose group (*n* = 80)	Risk ratio^a^ (95 % CI)	*p* value^#^
Clinical parameters at admission				
Gender M/F ratio	16/8	46/34	1.36 (0.64–2.87)	0.42
Age (years)	61 (47–71)	60 (49–64)		0.43
Age > 50 years	17 (71)	59 (74)	0.89 (0.46–1.93)	0.78
Age > 65 years	9 (38)	21 (44)	1.55 (0.77–3.14)	0.23
Duration of respiratory symptoms at diagnosis	7 (3–19)	10 (3–14)		0.87
Temperature (°C)	37.8 (37.3–38.8)	37.7 (37.0–38.3)		0.27
Respiratory rate (breaths per min)	24 (20–30)	23 (16–30)		0.33
Underlying condition				
HIV infection	5 (21)	12 (15)	1.35 (0.58–3.11)	0.50
Solid organ transplantation	6 (25)	27 (34)	0.72 (0.31–1.64)	0.42
Haematology malignancy	7 (29)^b^	25 (31)	0.93 (0.43–2.01)	0.85
Other	7 (29)	16 (20)	1.44 (0.68–3.07)	0.34
Laboratory results				
Leukocyte count (×10^9^/l)	7.2 (3.6–10.9)	7.2 (4.8–11.3)		0.91
Leukocyte count elevated (>ULN)	8 (33)	26 (33)	1.00 (0.48–2.13)	0.97
C-reactive protein (mg/l)	74 (36–153)	92 (48–194)		0.43
C-reactive protein >100 mg/l	10/21 (48)	28/61 (46)	1.05 (0.50–2.20)	0.89
Creatinine (µmol/l)	86 (74–134)	88 (64–147)		0.52
Creatinine elevated (>1.5 ULN)	5 (21)	20 (25)	0.82 (0.34–1.97)	0.65
Lactate dehydrogenase elevated (>1.5 ULN)	7/23 (30)	42/78 (54)	0.46 (0.21–1.03)	**0.048**
PaO_2_ (kPa)	7.9 (6.5–9.2)	7.9 (6.9–8.8)		0.84
Indication for steroid treatment [PaO_2_ (kPa) < 9.0]	11/16 (69)	48/61 (79)	0.67 (0.27–1.68)	0.40
Start steroid schedule at diagnosis	21/23 (91)	55/65 (85)	1.67 (0.44–6.17)	0.42
Outcome of diagnostic methods on BAL				
PCR positive	24 (100)^c^	76 (100)^d^	–	–
Microscopy positive	14/20 (70)	41/72 (57)	1.49 (0.66–3.72)	0.29
*C*_t_ value	33.7 (30.4–34.6)	34.1 (30.7–37.1)		0.74

Values represent median (IQR Q1–Q3) or no. (%)

*PCP* *Pneumocystis jirovecii* pneumonia, *CI* confidence interval, *HIV* human immunodeficiency virus, *ULN* upper limit of normal, *BAL* bronchoalveolar lavage, *PCR* polymerase chain reaction, *IQR* interquartile range

^#^ *p* value as calculated by Pearson Chi square test for bivariate variables and by Mann–Whitney *U* test or independent samples Student’s *t* test (according to distribution) for continuous variables

^a^Risk ratios are for risk of switch to low-dose treatment

^b^One patient in the step-down group with both non-Hodgkin lymphoma and a kidney transplantation

^c^In two cases, there was only a positive sputum PCR and no BAL performed

^d^In three cases, there was only a positive sputum PCR and no BAL performed; in four cases PCR was not performed, but microscopy was positive

### Mortality and relapse

The 30-day all-cause mortality was 13 % (14 patients). There was one death in the step-down group (4 %) versus 13 deaths (16 %) in the intermediate-dose group (*p* = 0.13, Table 3). The 14 patients with 30-day mortality are shown in Table 4. The patients died at a median of 14 days (IQR 9–18) after diagnosis of PCP. In two patients (14 %), the mortality was not related to PCP (1 patient with multi-organ failure and upper gastrointestinal bleeding and 1 patient with hypovolemic shock due to abdominal bleeding). In three patients (21 %), PCP contributed to mortality and in eight patients (57 %) death was attributable to PCP. One patient died of unknown cause. In the only patient who died in the step-down group, death was attributable to PCP.

**Table 3:** ICU admission, hospital stay, toxicity and outcome of patients with PCP

	Step-down group (*n* = 24)	Intermediate-dose group (*n* = 80)	Risk ratio^a^ (95 % CI)	*p* value^#^
ICU admission and hospital stay				
ICU admission	4 (17)	26 (33)	0.49 (0.18–1.32)	0.13
Length of hospitalization (days)	15 (9–24)	15 (8–33)		0.91
Toxicity				
(Pre-switch) in file reported TMP–SMX toxicity	5 (21)	4 (5)	2.78 (1.37–5.65)	**0.02**
Outcome				
30-day relapse rate	1 (4)	0	–	0.06
30-day mortality	1 (4)	13 (16)	0.28 (0.04–1.91)	0.13
100-day mortality	2 (8)	20 (25)	0.34 (0.09–1.33)	0.08

Values are median (IQR Q1–Q3) or no. (%)

*PCP* *Pneumocystis jirovecii* pneumonia, *CI* confidence interval, *ICU* intensive care unit, *IQR* interquartile range

^#^ *p* value as calculated by Pearson Chi square test for bivariate variables and by Mann–Whitney *U* test or independent samples Student’s *t* test (according to distribution) for continuous variables

^a^Risk ratios are for risk of switch to low-dose treatment

**Table 4:** Clinical characteristics, treatment and cause of death of 14 patients with fatal *Pneumocystis jirovecii* pneumonia

Patient	Age	Sex	Underlying condition	Duration of symptoms (days)	PaO_2_ (kPa)	Respiratory rate (bpm)	ICU admission (duration in days)	TMP–SMX regimen	Treatment duration at death (days)	Cause of death
1	36	V	Kidney transplantation	NA	6.6	NA	Yes (11)	ID	14	Respiratory failure
2	73	M	Pulmonary fibrosis	15	6.4	NA	Yes (16)	ID	18	Respiratory failure
3	86	M	Rheumatoid arthritis	14	6.6	24	Yes (7)	ID	8	Respiratory failure, renal failure
4	75	M	Kidney transplantation	14	7.8	24	Yes (6)	ID	13	Abdominal sepsis
5	63	M	Waldenstrom macroglobulinemia	14	8.2	24	Yes (4)	ID	5	Sepsis, respiratory failure, renal failure
6	53	V	Autoimmune hepatitis, RA	NA	8.4	40	Yes (14)	ID	14	Multi-organ failure, upper gastrointestinal bleeding
7	47	M	Metastasized rectal carcinoma	1	6.1	NA	No	ID	8	Respiratory failure
8	64	M	Interstitial lung disease	2	NA	30	Yes (12)	ID	14 + 6^a^	Respiratory failure
9	68	M	HIV	7	7.4	20	Yes (17)	ID	20	Respiratory failure
10	57	V	Autoimmune hepatitis	NA	NA	18	Yes (3)	ID	18	Hypovolemic shock due to abdominal bleeding
11	51	M	ALL, allogenic HCT	3	NA	20	No	SD	4	Respiratory failure
12	60	M	Pulmonary fibrosis, Crohn disease	2	5.1	30	Yes (16)	ID	14 + 3^a^	Respiratory failure
13	61	V	Lung carcinoma, Cushing	NA	8.7	NA	Yes (12)	ID	14	Abdominal sepsis, respiratory failure
14	83	M	CLL, hypogammaglobulinemia	2	5.7	22	No	ID	12	Unknown

*TMP*–*SMX* trimethoprim–sulfamethoxazole, *RA* rheumatoid arthritis, *HIV* human immunodeficiency virus, *ALL* acute lymphoblastic leukaemia, *HCT* haematopoietic cell transplantation, *CLL* chronic lymphocytic leukaemia, *NA* not available, *ICU* intensive care unit, *ID* intermediate-dose group, *SD* step-down group

^a^The patient died 6 days (respectively, 3 days) after 14 days of intermediate-dose TMP–SMX

The 100-day mortality was 8 % (2 patients) and 25 % (20 patients) for the step-down and intermediate-dose groups, respectively (*p* = 0.08).

There was one relapse in the low-dose group occurring 19 days after the initial episode. In this patient, the differential diagnosis was immune reconstitution inflammatory syndrome (IRIS) 10 days after starting combination antiretroviral therapy (cART) for a newly diagnosed HIV and PCP. In the second episode he recovered with steroid treatment only, although *P. jirovecii* was again detected in a fluid specimen from a second BAL.

Of note, the median length of hospitalization was 14.5 days in the step-down group versus 15 days in the intermediate-dose group (*p* = 0.91). ICU admission tended to be less frequent in the step-down group (17 vs. 33 %, *p* = 0.13). In a multivariate analysis adjusted for age and co-morbidity, CRP levels and the partial oxygen pressure at presentation were variables independently associated with later admission to the ICU (data not shown).

### Toxicity

Toxicity was more frequently reported in the step-down group, five patients in the step-down group (21 %) versus four patients in the intermediate-dose group (5 %). The reported toxicity included rash (5 patients), hepatotoxicity (2 patients), hyperkalaemia (2 patients) and renal insufficiency (1 patient). Toxicity was the reason to switch to low-dose TMP–SMX for the five patients in the step-down group after a median of 7 days of treatment. These five patients (4 with rash, 1 with elevated liver enzymes) continued their low-dose treatment with satisfactory results.

## Discussion

### TMP–SMX doses and treatment outcome of PCP

The results suggest that treatment of PCP with an intermediate dose of TMP–SMX has comparable efficacy as the recommended high-dose regimen. In addition, a step-down to low-dose TMP–SMX is likely an option for patients with a favourable clinical course and/or patients who already developed TMP–SMX-related toxicity. There was no increase in treatment failure or relapse in the step-down group.

Our experience with the intermediate dose of TMP–SMX complies with two previous studies in which a similar intermediate-dose regimen was used [11, 12]. Thomas et al. conducted a retrospective review of 73 HIV-infected patients with PCP treated with TMP 10 mg/kg/day and SMX 50 mg/kg/day. The relapse rate was 7 %. A low mortality rate of 7 % was reported, though ICU admission was only indicated in 12 % of cases. Bowden et al. conducted a randomized trial of 30 HIV-infected patients who received either TMP 20 mg/kg/day–SMX 100 mg/kg/day or TMP 10 mg/kg/day–SMX 50 mg/kg/day, with no differences in treatment failures. Mortality was not reported, but only patients with mild PCP were included. To the best of our knowledge, there is only one previous study that investigated a step-down regimen of TMP–SMX for the treatment of PCP [13]. Eeftinck Schattenkerk et al. retrospectively reviewed the case records of 50 patients with AIDS and PCP in the era prior to both cART and additional steroid therapy in the treatment of PCP. When drug intolerance developed, patients were switched to maintenance dose TMP–SMX (TMP 160 mg and SMX 800 mg o.d.). In this study, 20 patients were switched to maintenance-level TMP–SMX after an average of 9.6 days. There was no relapse in this group and overall survival was comparable with the group that received 2 weeks of high-dose TMP–SMX.

In contrast to this approach, the current protocol allowed patients with improving condition to switch also if no adverse events due to TMP–SMX were observed. Nevertheless, more adverse events were reported in the step-down group than in the intermediate-dose group, suggesting that the appearance of toxicity was an important incentive for physicians to pursue a switch to low-dose TMP–SMX. After the appearance of TMP–SMX toxicity, five patients were able to complete their treatment with low-dose TMP–SMX, thereby avoiding the use of alternative regimens like pentamidine or clindamycin plus primaquine.

Our study cohort contains many severe cases of PCP, i.e. ICU admission was required in 29 % of cases. The overall 30-day mortality rate of 13 % in our study is comparable to or even lower than the mortality rates of 12–18 %, reported from earlier studies. In these studies, a high dose of TMP–SMX was used for HIV-related PCP, with concomitant additional steroid treatment if indicated [7, 15–18]. Mortality rates for PCP in immunocompromised HIV-negative patients have been found to be even higher (35–50 %) [19–21].

### Clinical parameters and step-down to low-dose TMP–SMX

The current study did not identify the clinical characteristics at the time of diagnosis that were predictive of a successful switch to low-dose TMP–SMX. Lower levels of LDH were present in the step-down group, but this parameter was not sufficiently discriminative. Notably, clinical variables known to be predictive for an unfavourable outcome, e.g. age, partial oxygen pressure and high C-reactive protein (CRP) [22–24], were not different between the two treatment groups. In a separate analysis (data not shown), the correlation between mortality and high CRP as well as low partial oxygen pressure at presentation was confirmed in our cohort. Several factors may explain the fact that the clinical characteristics—at presentation—usually associated with a favourable outcome were not found to be associated with a switch to low-dose treatment. First, a subcategory of patients that continued treatment on intermediate-dose TMP–SMX might have done well also after switch to low-dose therapy. The opportunity to switch may simply have been missed by the attending physicians. Secondly, the decision to switch to low-dose therapy was made during the course of treatment and not at presentation. Reassessment of clinical variables at the time of consideration of decreasing the dose of TMP–SMX could more accurately disclose which clinical variables predict a successful step-down strategy.

### Pharmacology of TMP–SMX and treatment of PCP

Because of the difficulties of propagating *P. jirovecii* for a prolonged time in vitro, minimal inhibitory concentrations (MICs) and clinical breakpoints have not been obtained for TMP–SMX with regard to treatment of PCP [25–27]. Data from animal studies on this subject are available but concern different *Pneumocystis* species, e.g. *P. carinii* [4]. The results of these investigations may not be easily extrapolated to PCP in humans. TMP–SMX has excellent bioavailability, nearly 100 % after oral administration [28]. For trimethoprim, serum levels of 2 µg/ml are reached after ingestion of 960 mg of TMP–SMX, and remain stable for approximately 6 h, after which the concentration steadily falls [29]. The TMP to SMX concentration ratio in cotrimoxazole is 1:5, but in serum it reaches a concentration ratio of 1:20 and both compounds are bound to serum proteins for approximately two-thirds of their total concentration [30]. As a consequence, a dose of 960 mg cotrimoxazole b.i.d. would approximately result in effective (i.e. unbound) concentrations of 0.67 and 13.3 µg/ml of TMP and SMX, respectively, for 12 per 24 h. From in vitro studies, the estimates of the IC_90_ of SMX for *P. carinii* (i.e. rat *Pneumocystis*) were found to be 8 µg/ml (trophozoites) and 16 µg/ml (cysts) when combined with 0.5 µg/ml TMP [31]. Although results of such experiments have not been published for *P. jirovecii*, this suggests that effective killing of *P. jirovecii* for over 50 % of the time treated with cotrimoxazole 960 mg b.i.d. is expected. However, further in vitro study of *P. jirovecii* susceptibility is needed to provide a stronger pharmacological basis for the rationale of treatment with low-dose TMP–SMX. Due to good tissue penetration, concentrations of TMP and SMX in organs have frequently been found to be higher than serum concentrations [32, 33].

### Study limitations and strengths

Because of its non-randomized design, equal effectiveness or non-inferiority of the executed strategy cannot be claimed. The obvious confounding by indication also explains the higher mortality in the intermediate-dose group, and patients with a favourable clinical course would be more prevalent in the step-down group. In addition, toxicity was an important reason for the attending physician to switch to low-dose TMP–SMX, accounting for the high frequency of reported toxicity in the step-down group.

The study included a substantial number of patients and the clinical practice setting is representative of standard diagnostic workup and care for patients with PCP. Different groups of immunocompromised patients are equally represented in both groups. This emphasizes that the step-down approach was followed regardless of the underlying immunodeficiency of the patient. Therefore, differences between PCP in HIV and non-HIV infected patients probably do not impair the interpretation of the results [34, 35].

## Conclusions

Despite the observational design of the study, the results strongly suggest that treatment of PCP with intermediate-dose TMP–SMX has a similar efficacy compared to treatment with the recommended high-dose regimen. A randomized controlled trial would be necessary to provide clear evidence that both regimens are equally effective. Since the superiority of the high-dose regimen over an intermediate-dose or even over a low-dose regimen was never established by modern epidemiological standards in any clinical trial, the standard use of an intermediate-dose TMP–SMX as PCP therapy is—at the very least—open for discussion. The issue of lower frequency of severe toxic side effects, i.e. neutropenia, drug-induced hepatitis and rash, observed in patients who use an intermediate dose herein plays an important role.

In addition, a consecutive step-down strategy to low-dose TMP–SMX appears to be successful in selected patients, regardless of underlying disease and clinical characteristics at presentation. In case of development of toxicity during treatment with intermediate-dose TMP–SMX, our results confirm that most of these patients are able to complete treatment with low-dose TMP–SMX.

Further studies should focus on optimizing the balance between efficacy and toxicity of TMP–SMX by patient-tailored treatment of PCP.

## References

[1] Panel on Opportunistic Infections in HIV-Infected Adults and Adolescents. Guidelines for the prevention and treatment of opportunistic infections in HIV-infected adults and adolescents: recommendations from the Centers for Disease Control and Prevention, the National Institutes of Health, and the HIV Medicine Association of the Infectious Diseases Society of America. http://aidsinfo.nih.gov/contentfiles/lvguidelines/adult_oi.pdf. Accessed 18 June 2014.

[2] Walzer PD, Smulian AG. Pneumocystis species. In: Mandell GL, Bennett JE, Dolin R, editors. Mandell, Douglas, and Bennett’s principles and practice of infectious diseases, 7th ed. Philadelphia; 2010. p. 3377–90.

[3] Gilroy SA, Bennett NJ (2011). Pneumocystis pneumonia. Semin Respir Crit Care Med.

[4] Hughes WT, McNabb PC, Makres TD, Feldman S (1974). Efficacy of trimethoprim and sulfamethoxazole in the prevention and treatment of *Pneumocystis carinii* pneumonitis. Antimicrob Agents Chemother.

[5] Hughes WT, Feldman S, Sanyal SK (1975). Treatment of *Pneumocystis carinii* pneumonitis with trimethoprim–sulfamethoxazole. Can Med Assoc J.

[6] Wharton JM, Coleman DL, Wofsy CB, Luce JM, Blumenfeld W, Hadley WK, Ingram-Drake L, Volberding PA, Hopewell PC (1986). Trimethoprim–sulfamethoxazole or pentamidine for *Pneumocystis carinii* pneumonia in the acquired immunodeficiency syndrome. A prospective randomized trial. Ann Intern Med.

[7] Sattler FR, Frame P, Davis R, Nichols L, Shelton B, Akil B, Baughman R, Hughlett C, Weiss W, Boylen CT, van der Horst C, Black J, Powderly W, Steigbigel RT, Leedom JM, Masur H, Feinberg J (1994). Trimetrexate with leucovorin versus trimethoprim–sulfamethoxazole for moderate to severe episodes of *Pneumocystis carinii* pneumonia in patients with AIDS: a prospective, controlled multicenter investigation of the AIDS Clinical Trials Group Protocol 029/031. J Infect Dis.

[8] Klein NC, Duncanson FP, Lenox TH, Forszpaniak C, Sherer CB, Quentzel H, Nunez M, Suarez M, Kawwaff O, Pitta-Alvarez A, Freeman K, Wormser GP (1992). Trimethoprim–sulfamethoxazole versus pentamidine for *Pneumocystis carinii* pneumonia in AIDS patients: results of a large prospective randomized treatment trial. AIDS.

[9] Safrin S, Finkelstein DM, Feinberg J, Frame P, Simpson G, Wu A, Cheung T, Soeiro R, Hojczyk P, Black JR (1996). Comparison of three regimens for treatment of mild to moderate *Pneumocystis carinii* pneumonia in patients with AIDS. A double-blind, randomized, trial of oral trimethoprim–sulfamethoxazole, dapsone–trimethoprim, and clindamycin–primaquine. ACTG 108 Study Group. Ann Intern Med.

[10] Hughes W, Leoung G, Kramer F, Bozzette SA, Safrin S, Frame P, Clumeck N, Masur H, Lancaster D, Chan C, Lavelle J, Rosenstock J, Falloon J, Feinberg J, Lafon S, Rogers M, Sattler F (1993). Comparison of atovaquone (566C80) with trimethoprim–sulfamethoxazole to treat *Pneumocystis carinii* pneumonia in patients with AIDS. N Engl J Med.

[11] Thomas M, Rupali P, Woodhouse A, Ellis-Pegler R (2009). Good outcome with trimethoprim 10 mg/kg/day–sulfamethoxazole 50 mg/kg/day for *Pneumocystis jirovecii* pneumoniae in HIV infected patients. Scand J Infect Dis.

[12] Bowden FJ, Stewart K, Mashford L, Lucas CR (1991). A randomised, double blind trial of low dose versus high dose cotrimoxazole in the treatment of aids-related *Pneumocystis carinii* pneumonia. Aust N Z J Med.

[13] Eeftinck Schattenkerk JK, Lange JM, van Steenwijk RP, Danner SA (1990). Can the course of high dose cotrimoxazole for *Pneumocystis carinii* pneumonia in AIDS be shorter? A possible solution to the problem of cotrimoxazole toxicity. J Intern Med.

[14] Linssen CF, Jacobs JA, Beckers P, Templeton KE, Bakkers J, Kuijper EJ, Melchers WJ, Drent M, Vink C (2006). Inter-laboratory comparison of three different real-time PCR assays for the detection of *Pneumocystis jirovecii* in bronchoalveolar lavage fluid samples. J Med Microbiol.

[15] Benfield TL, Helweg-Larsen J, Bang D, Junge J, Lundgren JD (2001). Prognostic markers of short-term mortality in AIDS-associated *Pneumocystis carinii* pneumonia. Chest.

[16] Dworkin MS, Hanson DL, Navin TR (2001). Survival of patients with AIDS, after diagnosis of *Pneumocystis carinii* pneumonia, in the United States. J Infect Dis.

[17] Pulvirenti J, Herrera P, Venkataraman P, Ahmed N (2003). *Pneumocystis carinii* pneumonia in HIV-infected patients in the HAART era. AIDS Patient Care STDS.

[18] Rahdi S, Alexander T, Ukwu M, Saleh S, Morris A (2008). Outcome of HIV-associated Pneumocystis pneumonia in hospitalized patients from 2000 through 2003. BMC Infect Dis.

[19] Arend SM, Kroon FP, van’t Wout JW (1995). *Pneumocystis carinii* pneumonia in patients without AIDS, 1980 through 1993. An analysis of 78 cases. Arch Intern Med.

[20] Roblot F, Godet C, Le Moal G, Garo B, Faouzi Souala M, Dary M, De Gentile L, Gandji JA, Guimard Y, Lacroix C, Roblot P, Becq-Giraudon B (2002). Analysis of underlying diseases and prognosis factors associated with *Pneumocystis carinii* pneumonia in immunocompromised HIV-negative patients. Eur J Clin Microbiol Infect Dis.

[21] Sepkowitz KA, Brown AE, Telzak EE, Gottlieb S, Armstrong D (1992). *Pneumocystis carinii* pneumonia among patients without AIDS at a cancer hospital. JAMA.

[22] Armstrong-James D, Copas AJ, Walzer PD, Edwards SG, Miller RF (2011). A prognostic scoring tool for identification of patients at high and low risk of death from HIV-associated *Pneumocystis jirovecii* pneumonia. Int J STD AIDS.

[23] Sage EK, Noursadeghi M, Evans HE, Parker SJ, Copas AJ, Edwards SG, Miller RF (2010). Prognostic value of C-reactive protein in HIV-infected patients with *Pneumocystis jirovecii* pneumonia. Int J STD AIDS.

[24] Fei MW, Kim EJ, Sant CA, Jarlsberg LG, Davis JL, Swartzman A, Huang L (2009). Predicting mortality from HIV-associated Pneumocystis pneumonia at illness presentation: an observational cohort study. Thorax.

[25] Sloand E, Laughon B, Armstrong M, Bartlett MS, Blumenfeld W, Cushion M, Kalica A, Kovacs JA, Martin W, Pitt E, Pesanti EL, Richards F, Rose R, Walzer P (1993). The challenge of *Pneumocystis carinii* culture. J Eukaryot Microbiol.

[26] Thomas CF, Limper AH (2007). Current insights into the biology and pathogenesis of Pneumocystis pneumonia. Nat Rev Microbiol.

[27] Merali S, Frevert U, Williams JH, Chin K, Bryan R, Clarkson AB (1999). Continuous axenic cultivation of *Pneumocystis carinii*. Proc Natl Acad Sci USA.

[28] Chin TW, Vandenbroucke A, Fong IW (1995). Pharmacokinetics of trimethoprim–sulfamethoxazole in critically ill and non-critically ill AIDS patients. Antimicrob Agents Chemother.

[29] Bushby SR, Hitchings GH (1968). Trimethoprim, a sulphonamide potentiator. Br J Pharmacol Chemother.

[30] Bergan T, Brodwall EK (1976). The pharmacokinetic profile of co-trimoxazole. Scand J Infect Dis Suppl.

[31] Cirioni O, Giacometti A, Scalise G (1997). In-vitro activity of atovaquone, sulphamethoxazole and dapsone alone and combined with inhibitors of dihydrofolate reductase and macrolides against *Pneumocystis carinii*. J Antimicrob Chemother.

[32] Craig WA, Kunin CM (1973). Distribution of trimethoprim–sulfamethoxazole in tissues of rhesus monkeys. J Infect Dis.

[33] Dubar V, Lopez I, Gosset P, Aerts C, Voisin C, Wallaert B (1990). The penetration of co-trimoxazole into alveolar macrophages and its effect on inflammatory and immunoregulatory functions. J Antimicrob Chemother.

[34] Nuesch R, Bellini C, Zimmerli W (1999). *Pneumocystis carinii* pneumonia in human immunodeficiency virus (HIV)-positive and HIV-negative immunocompromised patients. Clin Infect Dis.

[35] Sepkowitz KA (2002). Opportunistic infections in patients with and patients without acquired immunodeficiency syndrome. Clin Infect Dis.

